# Regulation of Endotoxin Tolerance and Compensatory Anti-inflammatory Response Syndrome by Non-coding RNAs

**DOI:** 10.3389/fimmu.2018.02705

**Published:** 2018-11-20

**Authors:** Eleni Vergadi, Katerina Vaporidi, Christos Tsatsanis

**Affiliations:** ^1^Department of Paediatrics, Medical School, University of Crete, Heraklion, Greece; ^2^Department of Clinical Chemistry, Medical School, University of Crete, Heraklion, Greece; ^3^Department of Intensive Care Medicine, Medical School, University of Crete, Heraklion, Greece

**Keywords:** endotoxin tolerance, sepsis, immune suppression, microRNAs, non-coding RNAs, soluble mediators, lncRNAs

## Abstract

The onset and the termination of innate immune response must be tightly regulated to maintain homeostasis and prevent excessive inflammation, which can be detrimental to the organism, particularly in the context of sepsis. Endotoxin tolerance and compensatory anti-inflammatory response syndrome (CARS) describe a state of hypo-responsiveness characterized by reduced capacity of myeloid cells to respond to inflammatory stimuli, particularly those initiated by bacterial lipopolysaccharide (LPS). To achieve endotoxin tolerance, extensive reprogramming otherwise termed as “innate immune training”, is required that leads to both modifications of the intracellular components of TLR signaling and also to alterations in extracellular soluble mediators. Non-coding RNAs (ncRNAs) have been recognized as critical regulators of TLR signaling. Specifically, several microRNAs (miR-146, miR-125b, miR-98, miR-579, miR-132, let-7e and others) are induced upon TLR activation and reciprocally promote endotoxin tolerance and/or cross tolerance. Many other miRNAs have been also shown to negatively regulate TLR signaling. The long non-coding (lnc)RNAs (Mirt2, THRIL, MALAT1, lincRNA-21 and others) are also altered upon TLR activation and negatively regulate TLR signaling. Furthermore, the promotion or termination of myeloid cell tolerance is not only regulated by intracellular mediators but is also affected by other TLR-independent soluble signals that often achieve their effect via modulation of intracellular ncRNAs. In this article, we review recent evidence on the role of different ncRNAs in the context of innate immune cell tolerance and trained immunity, and evaluate their impact on immune system homeostasis.

## Introduction

The onset and termination of the host immune responses have to be tightly controlled; the initial burst of pro-inflammatory cytokines should be timely blunted to avoid overwhelming inflammatory responses causing tissue damage and secure homeostasis. Endotoxin tolerance is a crucial homeostatic mechanism that prevents from the excessive activation of innate immune responses upon sustained toll-like receptor (TLR) stimulation.

Endotoxin tolerance is defined as the reduced capacity of a cell to respond to gram(-) bacterial lipopolysaccharide (LPS) after an initial exposure to this stimulus (1, 2). Endotoxin tolerance is considered a type of “innate immune memory” (3), a condition describing tolerance to pathogens, characterized by innate immune hypo—responsiveness or “immune-paralysis”. It occurs as a result of persistent TLR stimulation not only from LPS but from other TLR agonists and even TLR-independent inflammatory mediators (1). The mechanism by which exposure to a particular TLR ligand or other inflammatory mediators such as cytokines reduces the inflammatory response to different TLR ligands is known as cross–tolerance and cytokine-induced tolerance, respectively (4–7), both being part of the innate immune system training (8, 9).

The phenotype of endotoxin tolerance and cross-tolerance has been extensively studied in monocytes and macrophages, even though the majority of innate immune cells develop tolerance to secondary TLR stimuli. These include dendritic cells, neutrophils, mast cells as well as endothelial and epithelial cells (10–14).

Endotoxin tolerance results to a shift of the cell phenotype from pro-inflammatory to anti-inflammatory (15). Endotoxin tolerant macrophages are reprogrammed to produce less tumor necrosis factor alpha (TNFα), interleukin (IL)-12 and IL-6 upon secondary stimulation and more anti-inflammatory cytokines such as IL-10 and transforming growth factor beta (TGFβ), compared to the levels produced from naive cells (16, 17). Furthermore, tolerant macrophages and dendritic cells downregulate human leukocyte antigen (HLA-DR) receptors thus have impaired capability for antigen presentation (16, 18). Similar phenotype is also described in cross-tolerance, but to a lesser extent (19). Endotoxin tolerant phenotype is long lasting but reversible in nature.

The clinical manifestation of endotoxin tolerance is recognized as Compensatory Anti-inflammatory Response Syndrome (CARS) (20). CARS represents the phase of immune “exhaustion” otherwise termed “immune paralysis”, that is observed in a subset of septic patients usually following the first phase of sepsis, known as Systemic Inflammatory Response Syndrome (SIRS) (21). Endotoxin tolerance explains CARS immunosuppression state in sepsis since blood leukocytes from septic patients exhibit similar phenotype to endotoxin tolerant cells; neutrophils and monocytes from septic patients are refractory to production of inflammatory mediators while they upregulate anti-inflammatory molecules when exposed to secondary TLR stimuli (1, 17, 22, 23). As a result, patients with CARS exhibit increased susceptibility to secondary infections (24).

The mechanism of innate immune cell tolerance and CARS are tightly regulated by complex molecular signatures in macrophages and other innate immune cells. These molecular pathways are controlled not only by modulation of intracellular signaling proteins and histone modifications but also by non-coding (ncRNAs), mostly microRNAs (miRNAs) and long ncRNAs (lncRNAs). In this article, we review recent evidence on the role of ncRNAs, regulated by TLR ligands or other TLR independent soluble signals, in the regulation of endotoxin tolerance and discuss their impact in the context of sepsis.

## TLR—dependent regulation of endotoxin tolerance via ncRNAs

Upon stimulation by pathogen- or danger-associated patterns, TLR mediate signals through two distinct adaptors pathways, myeloid differentiation factor 88 (MyD88) and TIR-domain-containing adapter-inducing interferon-β (TRIF). The MyD88 pathway employs interleukin-1 receptor-associated kinase (IRAK)1 and 4 kinases and TNF receptor-associated factor (TRAF)6 to activate nuclear factor κB (NFκB) and mitogen activated protein kinase (MAPK)/activator protein 1 (AP-1) signaling, promoting transcription of pro-inflammatory cytokines. Activation of TRIF pathway leads to janus kinase (JAK)/signal transducer and activator of transcription (STAT) and type I interferon activation and increases the expression of interferon-inducible genes (25, 26). In TLR4 tolerance, defects in TLR4 signaling have been observed at all levels, including receptor adaptors, signaling molecules, transcription factors, as well as, chromatin marks as histone modifications (1, 27).

The molecular signature of endotoxin tolerance involves downregulation of TLR4 expression, decreased recruitment of MyD88 or TRIF to TLR4, decreased activation of IRAK1/4 and diminished NFκB signaling via formation of the inactive p50 homodimers (1, 28). Additionally, negative regulatory molecules such as IRAK-M, A20, SH2 domain-containing inositol phosphatase 1 (SHIP1), Pellino-3, suppression of tumorigenicity 2 (ST2), suppression of cytokine signaling (SOCS)3 and SOCS1 are upregulated in endotoxin tolerant cells and inhibit the activation of TLR signaling (1, 28–33). However, during last two decades, an additional level of regulation through non-coding regulatory RNAs has been introduced.

### TLR dependent miRNAs that regulate endotoxin tolerance

MicroRNAs (miRNAs) are a large family of small noncoding RNAs (about 22 nucleotides in length) that regulate gene expression post-transcriptionally, by binding to the 3′-untranslated regions (UTRs) of target mRNAs (34). MiRNAs are recognized as key players in the regulation of endotoxin tolerance since multiple levels of the TLR signaling cascade are controlled by miRNAs (35, 36). At the stage of endotoxin tolerance, two LPS inducible miRNAs, miR-155 and miR-146α have been shown to be coordinately regulated via gene colocalization and transcription factor binding, contributing to the regulation of endotoxin tolerance (37). Indeed, miR-146α was the first miRNA described to promote tolerance (38, 39). MiR-146α is induced upon TLR activation in macrophages and its expression is further upregulated with LPS restimulation (17, 37). MiR-146α then targets IRAK1 and TRAF6, critical components downstream TLR signaling and its prolonged expression has been linked to endotoxin tolerance and cross—tolerance (19, 39–41). On the other hand, miR-155 inhibits the expression of the negative regulators SHIP1 and SOCS1 enhancing TLR signals, promotes TNFα translation and establishes a proinflammatory phenotype in macrophages (42–46). However, other studies show that miR-155 may exert negative regulation of pro-inflammatory mediators (47) (Table 1). Suppression of miR-155 in Akt1^−/−^ macrophages restored sensitivity and tolerance to LPS *in vitro* and *in vivo*, supporting its role in the regulation of endotoxin tolerance (43).

**Table 1:** List of the most prominent miRNAs implicated in the regulation of innate immune cell tolerance.

**MiRNA**	**Response to TLR signal**	**Target**	**Mechanism of action**	**References**
miR-146α	Induced	TRAF6, IRAK1, TLR2/4, Notch1	Targets TLR and TRAF6, IRAK1 in macrophages critical components downstream TLR signaling	(38, 48)
miR-146b	Induced	TRAF6 IRAK1 TLR4	Targets TLR and TRAF6, IRAK1 critical components downstream TLR signaling	(39, 49)
miR-155	Induced	SHIP, SOCS1 CEBP/β FADD, Ripk1	Inhibits the expression of the negative regulators of TLR signaling, SHIP1 and SOCS1. Promotes TNFα translation. Abrogates expression of anti-inflammatory genes in macrophages	(42–44)
		MyD88 TAB2, IKKe	Negative regulation of inflammatory cytokine production in macrophages and DCs	(50, 51)
miR-221	Induced	TNFα STAT1 STAT2	Promotes TNFα degradation. Induces tolerance via chromatin remodeling mediated by SWI/SNF (switch/sucrose non-fermentable) and STAT1/2 in macrophages	(3, 52)
miR-222	Induced	STAT1, STAT2	Induces tolerance via chromatin remodeling mediated by SWI/SNF (switch/sucrose non-fermentable) and STAT1 and 2 in macrophages	(3)
miR-132	Induced	IRAK4 p300	Responsible for inducing cross tolerance in monocytes/macrophages. Negative effect on the expression of interferon-stimulated genes and antiviral immunity in endothelial cells	(53, 54)
miR-21	Induced	PDCD4 MyD88,IRAK1 IL-12p35	Negative regulation of TLR4 signaling in monocytes. Inhibits the expression of MyD88 and IRAK1 during viral infection.	(55–58)
miR-579	Induced	TNFα	Negative regulation of TNFα translation in monocytes.	(52)
miR-125b	Induced	TNFα MyD88	Negative regulation of TNFα translation. Negatively regulate viral responses by targeting TLR2/MyD88 signaling in monocytes.	(42, 59)
miR-212	Induced	IRAK4	Sustained expression is responsible for inducing cross tolerance in monocytes/macrophages.	(54)
let-7e	Induced	TLR4	Negative regulation of TLR4 signaling in macrophages	(43)
let-7i	Suppressed	TLR4	Post-transcription regulation of TLR4 in epithelial cells	(60)
miR-124	Induced	TLR6, MyD88 TRAF6, TNFα	Negatively regulates TLR signaling in BCG infection in macrophages	(61)
miR-149	Suppressed	MyD88	Represses MyD88 translation in macrophages	(62)
miR-203	Induced	MyD88	Represses MyD88 translation in macrophages	(63)
miR-92a	Suppressed	MAPK4	Inhibits TLR4 —responses in macrophages	(64)
miR-210	Induced	NFκB1	Targets NFκB1 upon stimulation in macrophages	(65)
miR-9	Induced	NFκB1	Negative control of NFκB in monocytes	(66)
miR-718	Induced	PTEN	Down regulates TLR4, IRAK1, and NFκB in a negative feedback loop in macrophages	(67)
miR-98	Suppressed	IL-10	Targets IL-10 in macrophages	(68)

MiR-98 targets IL-10 in macrophages, a key cytokine for development of endotoxin tolerance; miR-98 is decreased by LPS in macrophages, thus failing to suppress IL-10 (68). The miRNAs miR-221, miR-579 and miR-125b are also significantly induced in endotoxin tolerant macrophages and lead to TNFα inhibition; miR-221 promotes TNFα degradation, whereas miR-579 and miR-125b block its translation (52). MiR-132 and miR-212 are also induced upon TLR2 stimulation and their sustained expression promotes cross tolerance (54). In a recent report, miR-221 and miR-222 were identified as regulators of the functional reprogramming of macrophages during LPS tolerization (3). MiR-221 and miR-222 were induced after prolonged LPS stimulation in mice and both promoted transcriptional silencing of a subset of pro-inflammatory genes via regulation of chromatin remodeling mediated by SWI/SNF (switch/sucrose non-fermentable) and STAT transcription factors (3).

However, there is a significant number of other miRNAs that have been shown to negatively regulate TLR signaling (Table 1). Among the aforementioned miRNAs, miR-146, miR-155, miR-221 and miR-222 have been extensively studied and appear to have a central role in the regulation of innate immune tolerance. In the context of sepsis, the levels of miR-146, miR-150, miR-221 and miR-222 among other miRNAs, are dysregulated in the peripheral blood leukocytes in sepsis patients and correlate with immunoparalysis and severity of the disease (3, 69–71), thus providing potential prognostic/diagnostic biomarkers.

### LncRNAs that contribute to endotoxin tolerance

Long noncoding RNAs (lncRNA) are regulatory RNAs that are over 200 nucleotides in length and do not encode proteins (72–74). LncRNAs are classified based on their site of action into *cis*-lncRNAs and *trans*-lncRNAs (nearby or remote to genes) and based on their relative position to target mRNAs, being exonic sense, intronic sense, antisense, bidirectional, and intergenic (75, 76). In contrast to miRNAs that have a clear role in promoting post-transcriptional regulation of gene expression, lncRNAs exhibit plethora of actions via transcriptional, post-transcriptional and translational regulation of gene expression as well as via controlling mRNA stability and promoting epigenetic changes (72, 75, 77–79).

LncRNAs have emerged as important regulators of innate immune responses and TLR signaling (74, 79–83). In response to LPS or other TLR stimuli, the lncRNAs expression pattern is altered and lcnRNAs have been shown to either promote or suppress pro-inflammatory responses (80, 84–86).

Several TLR-inducible lncRNAs limit excessive inflammatory responses by negatively regulating TLR signaling. The LPS-responsive lncRNAs Mirt2, THRIL, MALAT1, NKILA, lincRNA-21, and SeT have been shown to suppress expression of pro-inflammatory mediators including TNFα, the central cytokine for tolerance and CARS (Table 2). Mirt2 is expressed in macrophages and induced by LPS, negatively regulating TLR4 signaling; Mirt2 inhibits TRAF6 ubiquitination thus blocking NFκB and MARK activation and subsequent TNFα production (87). THRIL is another immuno-regulatory lncRNA that was found to interact with hnRNPL at the promoter region of the TNFα gene inducing TNFα expression (88). However, THRIL is downregulated upon TLR2 triggering indicating that THRIL suppression may be a protective feedback loop controlling TNFα levels and promoting cross–tolerance (88). The lncRNA MALAT1 has been found to negatively regulate TLR response via inhibition of NFκB; MALAT1 is upregulated in LPS-activated macrophages and interacts with NFκB in the nucleus, inhibiting LPS-induced expression of TNFα and IL-6 (89). Importantly, MALAT1 was found to be dysregulated in granulocytes from septic patients indicating its clinical importance in sepsis and CARS (104). Similar to MALAT1, NKILA is another lncRNA that regulates TLR4 signaling and restrains NFκB activation; NKILA is induced by LPS in tumor cells and interacts with the NFκB/IκB complex, preventing its phosphorylation by IKKs and subsequent NFκB activation (90). LincRNA-p21 is induced by LPS in fibroblasts and regulates NFκB activity in monocytes; lincRNA-p21 physically binds to RelA/p65 mRNA blocking translation of p65, resulting in inhibition of NFκB (94, 97, 98). Finally, the lncRNA SeT is expressed in macrophages in response to LPS and its homologous deletion results in biallelic TNFα expression and increase in TNFα levels (91). This finding suggests that lncRNA SeT suppresses expression of one of the two TNFα alleles early upon LPS stimulation (91).

**Table 2:** LcnRNAs that have been implicated in the regulation of innate immune cell tolerance.

**LncRNA**	**Response to TLR signal**	**Target**	**Mechanism of action**	**References**
Mirt2	Induced	TRAF6	Inhibits TRAF6 ubiquitination, NFκB and MARK activation and subsequent TNFα production in macrophages	(87)
THRIL	Suppressed	TNFα	Interacts with hnRNPL at the promoter of TNFα gene inducing TNFα expression in macrophages	(88)
MALAT1	Induced	NFκB	Interacts with nuclear NFκB, inhibits LPS-induced TNFα and IL-6 in macrophages	(89)
NKILA	Induced	NFακB/IκB	Interacts with NFκB/IκB complex in epithelial tumor cells, preventing its phosphorylation by IKKs and subsequent NFκB activation	(90)
SeT	Induced	TNFα	Suppresses expression of one of the two TNFα alleles early upon LPS stimuli in macrophages	(91)
Lnc-IL-17R	Induced	IL-6	Promotes H3K27 trimethylation, inhibits LPS-inducible inflammatory response genes (IL-6, chemokines) in macrophages/endothelial cells	(84)
IL7-AS	Induced	IL-6	IL7-AS suppression induces IL-6 in macrophages	(92)
lincRNA-EPS	Suppressed	NFκB	Binds to chromatin, regulates the nuclear ribonucleoprotein L (hnRNPL), and suppress pro-inflammatory genes in macrophages	(93)
lincRNA-Cox2	Induced	NFκB	Activates the NFκB –regulated late-primary inflammatory genes via interaction with hnRNP-A/B and hnRNP-A2/B1 in macrophages. In epithelial cells it represses TNFα-induced IL-12β transcription via recruitment of Mi-2/NuRD repressor complex to the IL-12β promoter	(80, 94–96)
LincRNA-p21	Induced	RelA/p65	Induced by TLR stimuli in fibroblasts. Physically binds to RelA/p65 mRNA blocking translation of p65 in monocytes	(97, 98)
lnc-DC	Induced	STAT3	Activates STAT3 by preventing SHIP1 mediated STAT3 dephosphorylation, resulting in reduced ability of dendritic cells to activate T cells	(99, 100)
NeST or Tmevpg1	Induced	IFN-γ	Alters H3K4 trimethylation in *IFN-γ* locus, upregulates IFN-γ expression in T cells and indirectly mitigates endotoxin tolerance	(101)
Lethe	Induced	RelA	Binds and inactivates RelA/p65 and decreases p65 binding at NFκB sites to restrict excessive inflammatory response in fibroblasts	(102)
PACER	Induced	p50	Interacts and sequesters excess p50 from COX2 promoter, activates COX2 in macrophages and epithelial cells	(103)
PCGEM1	Suppressed	unknown	TLR4 tolerisation reversed LPS-induced PCGEM1 suppression in macrophages	(79)
HOTTIP	Suppressed	unknown	TLR4 tolerisation reversed LPS-induced suppression of HOTTIP in macrophages	(79)

Additional lncRNAs have been shown to suppress pro-inflammatory mediators such as IL-6 but their effect on TNFα expression has not been evaluated (Table 2). Lnc-IL-17R is up-regulated significantly in response to TLR2 and TLR4 agonists, promoting H3K27 trimethylation and inhibiting LPS-inducible inflammatory response genes, such as IL-6, adhesion molecules, and chemokines (84). Similarly, the lncRNA IL7-AS (antisense) is induced by LPS in macrophages; knockdown of IL7-AS results in upregulation of IL-6 (92). Finally, lincRNA-EPS is expressed in macrophages and dendritic cells and was downregulated upon microbial infection, while gain-of-function experiments revealed that lincRNA-EPS binds to chromatin, regulates the nuclear ribonucleoprotein L (hnRNPL), thus suppressing LPS-induced pro-inflammatory genes (93). In addition to the lncRNAs outlined, lincRNA-Cox2 is another LPS inducible lncRNA that regulates hundreds of genes, but it appears to act both as an enhancer and as a suppressor of inflammation (80, 94, 95, 105). Finally, in a recent report, TLR4 tolerisation reversed LPS-induced suppression of PCGEM1 and HOTTIP lncRNAs and upregulated snaR lcnRNA, but further investigation is required to define the function of these lncRNAs in the context of tolerance (79).

It appears that the changes in the outlined lncRNAs significantly regulate TLR signaling toward TLR reprogramming. However, the majority of the above lncRNAs were not evaluated in endotoxin tolerant experimental setting per se since their expression and function was not evaluated upon secondary TLR stimulation. Also, their relative contribution to tolerant state in conjunction with several miRNAs, that were mentioned above and have an established central role in endotoxin tolerance, has not been studied yet. Further research is required to address the importance and the level of contribution of these lcnRNAs in endotoxin tolerance and/or cross tolerance.

## TLR-independent regulation of endotoxin tolerance via ncRNAs

Establishment of endotoxin tolerance and cross-tolerance is not strictly a result of excessive TLR signaling and subsequent induction of intracellular regulators. The magnitude and duration of the innate cell tolerance is also controlled by a plethora of TLR—independent soluble mediators.

### Soluble mediators in innate immune cell tolerance and their impact in ncRNAs

Cytokines such as IL-1β, IL-10, TGFβ, and TNFα are capable to induce cross-tolerance or cytokine-mediated tolerance initiating intracellular signals similar to those of TLR ligands (17, 106). Indeed, IL-10 and TGFβ are part of a negative feedback loop produced from activated macrophages acting in an autocrine and paracrine manner to promote tolerance and suppress secondary TLR responses. However, LPS priming provokes more sustained tolerance than IL-10 priming, since IL-10-primed monocytes rapidly recover and produce TNFα (107). Also, endogenous hormones, such as adiponectin and glucocorticoids blunt LPS-induced inflammation and promote anti-inflammatory responses (108, 109). In contrast, interferons such as interferon gamma (INF-γ) and α2-interferon, are known to abrogate endotoxin tolerance and restore induction of pro-inflammatory cytokines (110, 111).

The aforementioned soluble mediators have been reported to achieve their effect via modulation of intracellular ncRNAs. The capability of IL-1β priming to induce tolerance and cross-tolerance in monocytes and epithelial cells is mediated via the increase of miR-146α (6). IL-10 has been shown to promote miR-146b upregulation in human monocytes and its transcription is driven by STAT3, a transcription factor induced by IL-10 signals (49, 112). Similarly, TGFβ also promotes tolerance in human monocytes via upregulation of miR-146b driven by the transcription factor RUNX3 (112). Glucocorticoids and TGFβ have been shown to downregulate TLR4 signaling via induction of miR-511-5p, which targets TLR4 (113).

Stimulation with TNFα promotes TNFα–induced tolerance via regulation of ncRNAs. The lncRNA implicated in suppression of NFκB inflammatory response in fibroblasts upon TNFα stimulation is Lethe; Lethe binds and inactivates RelA/p65 and decreases p65 binding at NFκB sites (102). Moreover, upon TNFα stimulation, lincRNA-Cox2 is induced and promotes recruitment of the Mi-2/nucleosome remodeling and deacetylase (Mi-2/NuRD) repressor complex to the IL-12β promoter suppressing IL-12β expression (96).

IFN-γ is another mediator that enhances macrophage activation and reverses tolerance via regulation of ncRNAs. IFN-γ is known to inhibit miR-146b expression, a miRNA that contributes to endotoxin tolerance (112). Also, IFN-γ induces phosphatase and tensin homolog (PTEN) via downregulation of miR-3473b; MiR-3473b targets PTEN and promotes Akt/glycogen synthase kinase 3 signaling and IL-10 production (114). Furthermore, NeST, also known as Tmevpg1 or IFNgAS1, is a lncRNA located near the IFN-γ gene in both humans and mice and positively regulates expression of IFN-γ in T cells via histone modifications in IFN-γ locus (101).

### Soluble ncRNAs as modulators of endotoxin tolerance

Tissue injury leads to release of extracellular vehicles (EVs) that frequently include miRNAs (115–117). EVs are present in the circulation acting in a paracrine and endocrine manner and can modulate pro-inflammatory cytokine production contributing to a tolerogenic response (116). In addition freely circulating extracellular miRNAs may function as TLR agonists inducing tolerance (55, 118). EVs also promote tolerance in distant cells. For example, Treg derived exosomes deliver miR-150-5p and miR-142-3p to dendritic cells leading to the induction of LPS-induced IL-10 and suppression of LPS-induced IL-6, thus promoting tolerance (119).

## Conclusions

To conclude, it appears that a variety of TLR ligands, cytokines, and soluble mediators control endotoxin tolerance and cross-tolerance via the regulation of ncRNAs. However, there is a significant number of ncRNAs that are implicated in endotoxin tolerance and their relative importance and contribution in this process remains unknown. It is also unclear whether a level of interdependency among these ncRNAs exists and how their function may converge toward common pathways or potentially contradict each other. Further research is required to take into account the levels of contribution of each ncRNA in the context of innate immune tolerance and to highlight the ones that have the potential to develop into therapeutic tools for CARS, the clinical syndrome associated with innate immune tolerance.

## Author contributions

EV, KV, and CT reviewed the literature and drafted the manuscript.

### Conflict of interest statement

The authors declare that the research was conducted in the absence of any commercial or financial relationships that could be construed as a potential conflict of interest.

## References

[1] Biswas SK Lopez-Collazo E. Endotoxin tolerance: new mechanisms, molecules and clinical significance. Trend Immunol. (2009) 30:475–87. 10.1016/j.it.2009.07.009 19781994

[2] Pena OM Pistolic J Raj D Fjell CD Hancock RE. Endotoxin tolerance represents a distinctive state of alternative polarization (M2) in human mononuclear cells. J Immunol. (2011) 186:7243–54. 10.4049/jimmunol.1001952 21576504

[3] Seeley JJ Baker RG Mohamed G Bruns T Hayden MS Deshmukh SD. Induction of innate immune memory via microRNA targeting of chromatin remodelling factors. Nature (2018) 559:114–9. 10.1038/s41586-018-0253-5 29950719 PMC6044474

[4] Julian MW Strange HR Ballinger MN Hotchkiss RS Papenfuss TL Crouser ED. Tolerance and cross-tolerance following toll-like receptor (TLR)-4 and−9 activation are mediated by IRAK-M and modulated by IL-7 in murine splenocytes. PLoS ONE (2015) 10:e0132921. 10.1371/journal.pone.0132921 26218271 PMC4517781

[5] Lee HJ Kim KC Han JA Choi SS Jung YJ. The early induction of suppressor of cytokine signaling 1 and the downregulation of toll-like receptors 7 and 9 induce tolerance in costimulated macrophages. Mol Cell (2015) 38:26–32. 10.14348/molcells.2015.2136 25518931 PMC4314129

[6] Nahid MA Satoh M Chan EK. Interleukin 1beta-responsive microRNA-146a is critical for the cytokine-induced tolerance and cross-tolerance to toll-like receptor ligands. J Innate Immun. (2015) 7:428–40. 10.1159/000371517 25896300 PMC4485520

[7] Butcher SK O'Carroll CE Wells CA Carmody RJ. Toll-like receptors drive specific patterns of tolerance and training on restimulation of macrophages. Front Immunol. (2018). 9:933. 10.3389/fimmu.2018.00933 29867935 PMC5960718

[8] Novakovic B Habibi E Wang SY Arts RJW Davar R Megchelenbrink W. beta-Glucan reverses the epigenetic state of LPS-induced immunological tolerance. Cell (2016) 167:1354–68 e1314. 10.1016/j.cell.2016.09.034 27863248 PMC5927328

[9] Bekkering S Arts RJW Novakovic B Kourtzelis I van der Heijden C Li Y. Metabolic induction of trained immunity through the mevalonate pathway. Cell (2018) 172:135–146 e139. 10.1016/j.cell.2017.11.025 29328908

[10] Ogawa H Rafiee P Heidemann J Fisher PJ Johnson NA Otterson MF. Mechanisms of endotoxin tolerance in human intestinal microvascular endothelial cells. J Immunol. (2003) 170:5956–64. 10.4049/jimmunol.170.12.5956 12794122

[11] Saturnino SF Prado RO Cunha-Melo JR Andrade MV. Endotoxin tolerance and cross-tolerance in mast cells involves TLR4, TLR2 and FcepsilonR1 interactions and SOCS expression: perspectives on immunomodulation in infectious and allergic diseases. BMC Infect Dis. (2010) 10:240. 10.1186/1471-2334-10-240 20707930 PMC2930646

[12] Fekete T Szabo A Beltrame L Vivar N Pivarcsi A Lanyi A. Constraints for monocyte-derived dendritic cell functions under inflammatory conditions. Eur J Immunol. (2012) 42:458–69. 10.1002/eji.201141924 22057588

[13] Gunther J Petzl W Zerbe H Schuberth HJ Seyfert HM TLR ligands, but not modulators of histone modifiers, can induce the complex immune response pattern of endotoxin tolerance in mammary epithelial cells. Innate Immun. (2017) 23:155–64. 10.1177/1753425916681076 27913794 PMC5410871

[14] Koch SR Lamb FS Hellman J Sherwood ER Stark RJ. Potentiation and tolerance of toll-like receptor priming in human endothelial cells. Transl. Res. (2017) 180:53–67 e54. 10.1016/j.trsl.2016.08.001 27567430 PMC5253081

[15] O'Carroll C Fagan A Shanahan F Carmody RJ. Identification of a unique hybrid macrophage-polarization state following recovery from lipopolysaccharide tolerance. J Immunol. (2014) 192:427–36. 10.4049/jimmunol.1301722 24337373

[16] del Fresno C Garcia-Rio F Gomez-Pina V Soares-Schanoski A Fernandez-Ruiz I Jurado T Potent phagocytic activity with impaired antigen presentation identifying lipopolysaccharide-tolerant human monocytes: demonstration in isolated monocytes from cystic fibrosis patients. J Immunol. (2009) 182:6494–507. 10.4049/jimmunol.0803350 19414804

[17] Nahid MA Satoh M Chan EK. MicroRNA in TLR signaling and endotoxin tolerance. Cell Mol Immunol. (2011) 8:388–403. 10.1038/cmi.2011.26 21822296 PMC3618661

[18] Winkler MS Rissiek A Priefler M Schwedhelm E Robbe L Bauer A. Human leucocyte antigen (HLA-DR) gene expression is reduced in sepsis and correlates with impaired TNFalpha response: a diagnostic tool for immunosuppression? PLoS ONE (2017) 12:e0182427. 10.1371/journal.pone.0182427 28771573 PMC5542660

[19] Nahid MA Satoh M Chan EK. Mechanistic role of microRNA-146a in endotoxin-induced differential cross-regulation of TLR signaling. J Immunol. (2011) 186:1723–34. 10.4049/jimmunol.1002311 21178010 PMC3608687

[20] Adib-Conquy M Cavaillon JM. Compensatory anti-inflammatory response syndrome. Thromb Haemost. (2009) 101:36–47. 10.1160/TH08-07-0421 19132187

[21] Ward NS Casserly B Ayala A. The compensatory anti-inflammatory response syndrome (CARS) in critically ill patients. Clin Chest Med. (2008) 29:617–25. 10.1016/j.ccm.2008.06.010 18954697 PMC2786900

[22] McCall CE Grosso-Wilmoth LM LaRue K Guzman RN Cousart SL. Tolerance to endotoxin-induced expression of the interleukin-1 beta gene in blood neutrophils of humans with the sepsis syndrome. J Clin Invest. (1993) 91:853–61. 10.1172/JCI116306 7680670 PMC288037

[23] Wheeler DS Lahni PM Denenberg AG Poynter SE Wong HR Cook JA. Induction of endotoxin tolerance enhances bacterial clearance and survival in murine polymicrobial sepsis. Shock (2008) 30:267–73. 10.1097/shk.0b013e318162c190 18197145 PMC2754132

[24] Chen WH. Turning down the heat: the potential role of RIP140 in inflammation. Cell Mol Immunol. (2012) 9:195–6. 10.1038/cmi.2012.9 22504951 PMC4012850

[25] Yamamoto M Sato S Hemmi H Hoshino K Kaisho T Sanjo H. Role of adaptor TRIF in the MyD88-independent toll-like receptor signaling pathway. Science (2003) 301:640–3. 10.1126/science.1087262 12855817

[26] Biswas SK Tergaonkar V Myeloid differentiation factor 88-independent Toll-like receptor pathway: sustaining inflammation or promoting tolerance? Int J Biochem Cell Biol. (2007) 39:1582–92. 10.1016/j.biocel.2007.04.021 17572133

[27] Perkins DJ Patel MC Blanco JC Vogel SN. Epigenetic mechanisms governing innate inflammatory responses. J Interferon Cytokine Res. (2016) 36:454–61. 10.1089/jir.2016.0003 27379867 PMC4931723

[28] Carmody RJ Ruan Q Palmer S Hilliard B Chen YH. Negative regulation of toll-like receptor signaling by NF-kappaB p50 ubiquitination blockade. Science (2007) 317:675–8. 10.1126/science.1142953 17673665

[29] Kobayashi K Hernandez LD Galan JE Janeway CAJr Medzhitov R Flavell RA. IRAK-M is a negative regulator of Toll-like receptor signaling. Cell (2002) 110:191–202. 10.1016/S0092-8674(02)00827-9 12150927

[30] Sly LM Rauh MJ Kalesnikoff J Song CH Krystal G. LPS-induced upregulation of SHIP is essential for endotoxin tolerance. Immunity (2004) 21:227–39. 10.1016/j.immuni.2004.07.010 15308103

[31] Hoogerwerf JJ Leendertse M Wieland CW de Vos AF de Boer JD Florquin S. Loss of suppression of tumorigenicity 2 (ST2) gene reverses sepsis-induced inhibition of lung host defense in mice. Am J Res Crit Care Med. (2011) 183:932–40. 10.1164/rccm.201006-0934OC 20959556

[32] Xiong Y Medvedev AE. Induction of endotoxin tolerance *in vivo* inhibits activation of IRAK4 and increases negative regulators IRAK-M, SHIP-1, and A20. J Leukocyte Biol. (2011) 90:1141–8. 10.1189/jlb.0611273 21934070 PMC3236548

[33] Murphy MB Xiong Y Pattabiraman G Manavalan TT Qiu F Medvedev AE. Pellino-3 promotes endotoxin tolerance and acts as a negative regulator of TLR2 and TLR4 signaling. J Leukocyte Biol. (2015) 98:963–74. 10.1189/jlb.2VMA0515-229RR 26310831 PMC4661042

[34] Bartel DP. MicroRNAs: target recognition and regulatory functions. Cell (2009) 136:215–33. 10.1016/j.cell.2009.01.002 19167326 PMC3794896

[35] Quinn EM Wang J Redmond HP. The emerging role of microRNA in regulation of endotoxin tolerance. J Leukocyte Biol. (2012) 91:721–7. 10.1189/jlb.1111571 22389313

[36] He X Jing Z Cheng G. MicroRNAs: new regulators of Toll-like receptor signalling pathways. BioMed Res Int. (2014) 2014:945169. 10.1155/2014/945169 24772440 PMC3977468

[37] Doxaki C Kampranis SC Eliopoulos AG Spilianakis C Tsatsanis C. Coordinated regulation of miR-155 and miR-146a genes during induction of endotoxin tolerance in macrophages. J Immunol. (2015) 195:5750–61. 10.4049/jimmunol.1500615 26538391

[38] Nahid MA Pauley KM Satoh M Chan E MiR-146a is critical for endotoxin-induced tolerance: implications in innate immunity. J Biol Chem. (2009) 284:34590–9. 10.1074/jbc.M109.056317 19840932 PMC2787321

[39] Taganov KD Boldin MP Chang KJ Baltimore D. NF-kappaB-dependent induction of microRNA miR-146, an inhibitor targeted to signaling proteins of innate immune responses. Proc Nat Acad Sci USA. (2006) 103:12481–6. 10.1073/pnas.0605298103 16885212 PMC1567904

[40] Vergadi E Vaporidi K Theodorakis EE Doxaki C Lagoudaki E Ieronymaki E. Akt2 deficiency protects from acute lung injury via alternative macrophage activation and miR-146a induction in mice. J Immunol. (2014) 192:394–406. 10.4049/jimmunol.1300959 24277697

[41] El Gazzar M Church A Liu T McCall CE MicroRNA-146a regulates both transcription silencing and translation disruption of TNF-alpha during TLR4-induced gene reprogramming. J Leukocyte Biol. (2011) 90:509–19. 10.1189/jlb.0211074 21562054 PMC3157899

[42] Tili E Michaille JJ Cimino A Costinean S Dumitru CD Adair B. Modulation of miR-155 and miR-125b levels following lipopolysaccharide/TNF-alpha stimulation and their possible roles in regulating the response to endotoxin shock. J Immunol. (2007) 179:5082–9. 10.4049/jimmunol.179.8.5082 17911593

[43] Androulidaki A Iliopoulos D Arranz A Doxaki C Schworer S Zacharioudaki V. The kinase Akt1 controls macrophage response to lipopolysaccharide by regulating microRNAs. Immunity (2009) 31:220–31. 10.1016/j.immuni.2009.06.024 19699171 PMC2865583

[44] O'Connell RM Chaudhuri AA Rao DS Baltimore D. Inositol phosphatase SHIP1 is a primary target of miR-155. Proc Nat Acad Sci USA. (2009) 106:7113–8. 10.1073/pnas.0902636106 19359473 PMC2678424

[45] Arranz A Doxaki C Vergadi E Martinez de la Torre Y Vaporidi K Lagoudaki ED. Akt1 and Akt2 protein kinases differentially contribute to macrophage polarization. Proc Nat Acad Sci USA. (2012) 109:9517–22. 10.1073/pnas.1119038109 22647600 PMC3386059

[46] Sun X Sun J Shao X Feng J Yan J Qin Y. Inhibition of microRNA-155 modulates endotoxin tolerance by upregulating suppressor of cytokine signaling 1 in microglia. Exp Therap Med. (2018) 15:4709–16. 10.3892/etm.2018.6032 29805490 PMC5952101

[47] Zhou R O'Hara SP Chen XM. MicroRNA regulation of innate immune responses in epithelial cells. Cell Mol Immunol. (2011) 8:371–9. 10.1038/cmi.2011.19 21725335 PMC4012889

[48] Bai Y Qian C Qian L Ma F Hou J Chen Y. Integrin CD11b negatively regulates TLR9-triggered dendritic cell cross-priming by upregulating microRNA-146a. J Immunol. (2012) 188:5293–02. 10.4049/jimmunol.1102371 22551553

[49] Curtale G Mirolo M Renzi TA Rossato M Bazzoni F Locati M. Negative regulation of Toll-like receptor 4 signaling by IL-10-dependent microRNA-146b. Proc Nat Acad Sci USA. (2013) 110:11499–504. 10.1073/pnas.1219852110 23798430 PMC3710884

[50] Ceppi M Pereira PM Dunand-Sauthier I Barras E Reith W Santos MA. MicroRNA-155 modulates the interleukin-1 signaling pathway in activated human monocyte derived dendritic cells. Pro Nat Acad Sci USA (2009) 106:2735–40. 10.1073/pnas.0811073106 19193853 PMC2650335

[51] Tang B Xiao B Liu Z Li N Zhu ED Li BS. Identification of MyD88 as a novel target of miR-155, involved in negative regulation of Helicobacter pylori-induced inflammation. FEBS Lett. (2010) 584:1481–6. 10.1016/j.febslet.2010.02.063 20219467

[52] El Gazzar M McCall CE MicroRNAs distinguish translational from transcriptional silencing during endotoxin tolerance. J Biol Chem. (2010) 285:20940–51. 10.1074/jbc.M110.115063 20435889 PMC2898346

[53] Lagos D Pollara G Henderson S Gratrix F Fabani M Milne RS miR-132 regulates antiviral innate immunity through suppression of the p300 transcriptional coactivator. Nat. Cell Biol. (2010) 12:513–9. 10.1038/ncb2054 20418869

[54] Nahid MA Yao B Dominguez-Gutierrez PR Kesavalu L Satoh M Chan EK. Regulation of TLR2-mediated tolerance and cross-tolerance through IRAK4 modulation by miR-132 and miR-212. J Immunol. (2013) 190:1250–63. 10.4049/jimmunol.1103060 23264652 PMC3552145

[55] Chen X Liang H Zhang J Zen K Zhang CY. micro*RNA*s are ligands of Toll-like receptors. RNA (2013) 19:737–9. 10.1261/rna.036319.112 23554231 PMC3683908

[56] Sheedy FJ Palsson-McDermott E Hennessy EJ Martin C O'Leary JJ Ruan Q. Negative regulation of TLR4 via targeting of the proinflammatory tumor suppressor PDCD4 by the microRNA miR-21. Nat. Immunol. (2010) 11:141–7. 10.1038/ni.1828 19946272

[57] Chen Y Chen J Wang H Shi J Wu K Liu S. HCV-induced miR-21 contributes to evasion of host immune system by targeting MyD88 and IRAK1. PLoS Pathog. (2013) 9:e1003248. 10.1371/journal.ppat.1003248 23633945 PMC3635988

[58] Wu Z Lu H Sheng J Li L. Inductive microRNA-21 impairs anti-mycobacterial responses by targeting IL-12 and Bcl-2. FEBS Lett. (2012) 586:2459–67. 10.1016/j.febslet.2012.06.004 22710123

[59] Peng C Wang H Zhang WJ Jie SH Tong QX Lu MJ. Inhibitory effect of miR-125b on hepatitis C virus core protein-induced TLR2/MyD88 signaling in THP-1 cells. World J Gastroenterol. (2016) 22:4354–61. 10.3748/wjg.v22.i17.4354 27158204 PMC4853693

[60] Chen XM Splinter PL O'Hara SP LaRusso NF. A cellular micro-RNA, let-7i, regulates Toll-like receptor 4 expression and contributes to cholangiocyte immune responses against *Cryptosporidium parvum* infection. J Biol Chem. (2007) 282:28929–38. 10.1074/jbc.M702633200 17660297 PMC2194650

[61] Ma C Li Y Li M Deng G Wu X Zeng J. microRNA-124 negatively regulates TLR signaling in alveolar macrophages in response to mycobacterial infection. Mol Immunol. (2014) 62:150–8. 10.1016/j.molimm.2014.06.014 24995397

[62] Xu G Zhang Z Xing Y Wei J Ge Z Liu X. MicroRNA-149 negatively regulates TLR-triggered inflammatory response in macrophages by targeting MyD88. J Cell Biochem. (2014) 115:919–27. 10.1002/jcb.24734 24375488

[63] Wei J Huang X Zhang Z Jia W Zhao Z Zhang Y. MyD88 as a target of microRNA-203 in regulation of lipopolysaccharide or Bacille Calmette-Guerin induced inflammatory response of macrophage RAW264.7 cells. Mol Immunol. (2013) 55:303–9. 10.1016/j.molimm.2013.03.004 23522925

[64] Lai L Song Y Liu Y Chen Q Han Q Chen W. MicroRNA-92a negatively regulates Toll-like receptor (TLR)-triggered inflammatory response in macrophages by targeting MKK4 kinase. J Biol Chem. (2013) 288:7956–67. 10.1074/jbc.M112.445429 23355465 PMC3597832

[65] Qi J Qiao Y Wang P Li S Zhao W Gao C. microRNA-210 negatively regulates LPS-induced production of proinflammatory cytokines by targeting NF-kappaB1 in murine macrophages. FEBS Lett. (2012) 586:1201–7. 10.1016/j.febslet.2012.03.011 22575656

[66] Bazzoni F Rossato M Fabbri M Gaudiosi D Mirolo M Mori L. Induction and regulatory function of miR-9 in human monocytes and neutrophils exposed to proinflammatory signals. Proc Nat Acad Sci USA. (2009) 106:5282–7. 10.1073/pnas.0810909106 19289835 PMC2664036

[67] Kalantari P Harandi OF Agarwal S Rus F Kurt-Jones EA Fitzgerald KA. miR-718 represses proinflammatory cytokine production through targeting phosphatase and tensin homolog (PTEN). J Biol Chem. (2017) 292:5634–44. 10.1074/jbc.M116.749325 28209713 PMC5392559

[68] Liu Y Chen Q Song Y Lai L Wang J Yu H. MicroRNA-98 negatively regulates IL-10 production and endotoxin tolerance in macrophages after LPS stimulation. FEBS Lett. (2011) 585:1963–8. 10.1016/j.febslet.2011.05.029 21609717

[69] Vasilescu C Rossi S Shimizu M Tudor S Veronese A Ferracin M. MicroRNA fingerprints identify miR-150 as a plasma prognostic marker in patients with sepsis. PLoS ONE (2009) 4:e7405. 10.1371/journal.pone.0007405 19823581 PMC2756627

[70] Wang JF Yu ML Yu G Bian JJ Deng XM Wan XJ. Serum miR-146a and miR-223 as potential new biomarkers for sepsis. Biochem Biophys Res Commun. (2010) 394:184–8. 10.1016/j.bbrc.2010.02.145 20188071

[71] Essandoh K Fan GC. Role of extracellular and intracellular microRNAs in sepsis. Biochim. et Biophy. Acta (2014) 1842:2155–62. 10.1016/j.bbadis.2014.07.021 25086335 PMC4188718

[72] Rinn JL Chang HY. Genome regulation by long noncoding RNAs. Ann Rev Biochem. (2012) 81:145–66. 10.1146/annurev-biochem-051410-092902 22663078 PMC3858397

[73] Guttman M Russell P Ingolia NT Weissman JS Lander ES Ribosome profiling provides evidence that large noncoding RNAs do not encode proteins. Cell (2013) 154:240–51. 10.1016/j.cell.2013.06.009 23810193 PMC3756563

[74] Carpenter S. Long noncoding RNA: Novel links between gene expression and innate immunity. Virus Res. (2016) 212:137–45. 10.1016/j.virusres.2015.08.019 26362525

[75] Mercer TR Dinger ME Mattick JS. Long non-coding RNAs: insights into functions. Nat Rev Genet. (2009) 10:155–9. 10.1038/nrg2521 19188922

[76] Meng XY Luo Y Anwar MN Sun Y Gao Y Zhang H. Long Non-coding RNAs: emerging and versatile regulators in host-virus interactions. Front Immunol. (2017) 8:1663. 10.3389/fimmu.2017.01663 29234324 PMC5712331

[77] Magistri M Faghihi MA St Laurent G IIIrd Wahlestedt C. Regulation of chromatin structure by long noncoding RNAs: focus on natural antisense transcripts. Trends Genet. (2012) 28:389–96. 10.1016/j.tig.2012.03.013 22541732 PMC3768148

[78] Ulitsky I Bartel DP. lincRNAs: genomics, evolution, and mechanisms. Cell (2013) 154:26–46. 10.1016/j.cell.2013.06.020 23827673 PMC3924787

[79] Murphy MB Medvedev AE. Long noncoding RNAs as regulators of Toll-like receptor signaling and innate immunity. J Leukocyte Biol. (2016) 99:839–50. 10.1189/jlb.2RU1215-575R 26965636 PMC6608019

[80] Carpenter S Aiello D Atianand MK Ricci EP Gandhi P Hall LL. A long noncoding RNA mediates both activation and repression of immune response genes. Science (2013) 341:789–92. 10.1126/science.1240925 23907535 PMC4376668

[81] Atianand MK Fitzgerald KA. Long non-coding RNAs and control of gene expression in the immune system. Trends Mol Med. (2014) 20:623–31. 10.1016/j.molmed.2014.09.002 25262537 PMC4252818

[82] Heward JA Lindsay MA. Long non-coding RNAs in the regulation of the immune response. Trends Immunol. (2014) 35:408–19. 10.1016/j.it.2014.07.005 25113636 PMC7106471

[83] Carpenter S. Determining the function of long noncoding RNA in innate immunity. Methods Mole Biol. (2016) 1390:183–95. 10.1007/978-1-4939-3335-8_12 26803630

[84] Cui H Xie N Tan Z Banerjee S Thannickal VJ Abraham E. The human long noncoding RNA lnc-IL7R regulates the inflammatory response. Eur J Immunol. (2014) 44:2085–95. 10.1002/eji.201344126 24723426 PMC4107034

[85] NE II Heward JA Roux B Tsitsiou E Fenwick PS Lenzi L Long non-coding RNAs and enhancer RNAs regulate the lipopolysaccharide-induced inflammatory response in human monocytes. Nat Commun. (2014) 5:3979 10.1038/ncomms4979 24909122 PMC4061460

[86] Mao AP Shen J Zuo Z. Expression and regulation of long noncoding RNAs in TLR4 signaling in mouse macrophages. BMC Genom. (2015) 16:45. 10.1186/s12864-015-1270-5 25652569 PMC4320810

[87] Du M Yuan L Tan X Huang D Wang X Zheng Z. The LPS-inducible lncRNA Mirt2 is a negative regulator of inflammation. Nat Commun. (2017) 8:2049. 10.1038/s41467-017-02229-1 29230038 PMC5725456

[88] Li Z Chao TC Chang KY Lin N Patil VS Shimizu C. The long noncoding RNA THRIL regulates TNFalpha expression through its interaction with hnRNPL. Proc Nat Acad Sci USA. (2014) 111:1002–7. 10.1073/pnas.1313768111 24371310 PMC3903238

[89] Zhao G Su Z Song D Mao Y Mao X. The long noncoding RNA MALAT1 regulates the lipopolysaccharide-induced inflammatory response through its interaction with NF-kappaB. FEBS Lett. (2016) 590:2884–95. 10.1002/1873-3468.12315 27434861

[90] Liu B Sun L Liu Q Gong C Yao Y Lv X. A cytoplasmic NF-kappaB interacting long noncoding RNA blocks IkappaB phosphorylation and suppresses breast cancer metastasis. Cancer Cell (2015) 27:370–81. 10.1016/j.ccell.2015.02.004 25759022

[91] Stathopoulou C Kapsetaki M Stratigi K Spilianakis C. Long non-coding RNA SeT and miR-155 regulate the Tnfalpha gene allelic expression profile. PLoS ONE (2017) 12:e0184788. 10.1371/journal.pone.0184788 28910376 PMC5599032

[92] Roux BT Heward JA Donnelly LE Jones SW Lindsay MA. Catalog of differentially expressed long non-coding RNA following activation of human and mouse innate immune response. Front Immunol. (2017) 8:1038. 10.3389/fimmu.2017.01038 28900427 PMC5581803

[93] Atianand MK Hu W Satpathy AT Shen Y Ricci EP Alvarez-Dominguez JR. A long noncoding RNA lincRNA-EPS acts as a transcriptional brake to restrain inflammation. Cell (2016) 165:1672–85. 10.1016/j.cell.2016.05.075 27315481 PMC5289747

[94] Mathy NW Chen XM. Long non-coding RNAs (lncRNAs) and their transcriptional control of inflammatory responses. J Biol Chem. (2017) 292:12375–82. 10.1074/jbc.R116.760884 28615453 PMC5535013

[95] Guttman M Amit I Garber M French C Lin MF Feldser D. Chromatin signature reveals over a thousand highly conserved large non-coding RNAs in mammals. Nature (2009) 458:223–7. 10.1038/nature07672 19182780 PMC2754849

[96] Tong Q Gong AY Zhang XT Lin C Ma S Chen J. LincRNA-Cox2 modulates TNF-alpha-induced transcription of Il12b gene in intestinal epithelial cells through regulation of Mi-2/NuRD-mediated epigenetic histone modifications. FASEB J. (2016) 30:1187–97. 10.1096/fj.15-279166 26578685 PMC4750408

[97] Spurlock CFIIIrd Tossberg JT Matlock BK Olsen NJ Aune TM. Methotrexate inhibits NF-kappaB activity via long intergenic (noncoding) RNA-p21 induction. Arthrit Rheumatol. (2014) 66:2947–57. 10.1002/art.38805 25077978 PMC4211976

[98] Zhou WQ Wang P Shao QP Wang J. Lipopolysaccharide promotes pulmonary fibrosis in acute respiratory distress syndrome (ARDS) via lincRNA-p21 induced inhibition of Thy-1 expression. Mol Cell Biochem. (2016) 419:19–28. 10.1007/s11010-016-2745-7 27392907

[99] Wang P Xue Y Han Y Lin L Wu C Xu S. The STAT3-binding long noncoding RNA lnc-DC controls human dendritic cell differentiation. Science (2014) 344:310–3. 10.1126/science.1251456 24744378

[100] Zhuang L Tian J Zhang X Wang H Huang C. Lnc-DC regulates cellular turnover and the HBV-induced immune response by TLR9/STAT3 signaling in dendritic cells. Cell Mol Biol Lett. (2018) 23:43. 10.1186/s11658-018-0108-y 30202418 PMC6122708

[101] Gomez JA Wapinski OL Yang YW Bureau JF Gopinath S Monack DM. The NeST long ncRNA controls microbial susceptibility and epigenetic activation of the interferon-gamma locus. Cell (2013) 152:743–54. 10.1016/j.cell.2013.01.015 23415224 PMC3577098

[102] Rapicavoli NA Qu K Zhang J Mikhail M Laberge RM Chang HY. A mammalian pseudogene lncRNA at the interface of inflammation and anti-inflammatory therapeutics. eLife (2013) 2:e00762. 10.7554/eLife.00762 23898399 PMC3721235

[103] Krawczyk M Emerson BM. p50-associated COX-2 extragenic RNA (PACER) activates COX-2 gene expression by occluding repressive NF-kappaB complexes. eLife (2014) 3:e01776. 10.7554/eLife.01776 24843008 PMC4017649

[104] Pellegrina D Severino P Barbeiro HV de Souza HP Machado MCC Pinheiro-da-Silva F. Insights into the function of long noncoding RNAs in sepsis revealed by gene co-expression network analysis. Non-Coding RNA (2017) 3:5. 10.3390/ncrna3010005 29657277 PMC5831999

[105] Ramirez-Carrozzi VR Nazarian AA Li CC Gore SL Sridharan R Imbalzano AN. Selective and antagonistic functions of SWI/SNF and Mi-2beta nucleosome remodeling complexes during an inflammatory response. Gen Dev. (2006) 20:282–96. 10.1101/gad.1383206 16452502 PMC1361700

[106] Mia S Warnecke A Zhang XM Malmstrom V Harris RA. An optimized protocol for human M2 macrophages using M-CSF and IL-4/IL-10/TGF-beta yields a dominant immunosuppressive phenotype. Scan J Immunol. (2014) 79:305–14. 10.1111/sji.12162 24521472 PMC4282403

[107] Wolk K Docke W von Baehr V Volk H Sabat R. Comparison of monocyte functions after LPS- or IL-10-induced reorientation: importance in clinical immunoparalysis. Pathobiology (1999) 67:253–6. 10.1159/000028104. 10725796

[108] Turner JJ Smolinska MJ Sacre SM Foxwell BM. Induction of TLR tolerance in human macrophages by adiponectin: does LPS play a role? Scan J Immunol. (2009) 69:329–36. 10.1111/j.1365-3083.2008.02224.x 19284497

[109] Li CC Munitic I Mittelstadt PR Castro E Ashwell JD. Suppression of dendritic cell-derived IL-12 by endogenous glucocorticoids is protective in LPS-induced sepsis. PLoS Biol. (2015) 13:e1002269. 10.1371/journal.pbio.1002269 26440998 PMC4595142

[110] Chen J Ivashkiv LB. IFN-gamma abrogates endotoxin tolerance by facilitating Toll-like receptor-induced chromatin remodeling. Proc Nat Acad Sci USA. (2010) 107:19438–43. 10.1073/pnas.1007816107 20974955 PMC2984206

[111] Shi L Song L Maurer K Sharp J Zhang Z Sullivan KE. Endotoxin tolerance in monocytes can be mitigated by alpha2-interferon. J Leukocyte Biol. (2015) 98:651–9. 10.1189/jlb.4A0914-450RR 26206900 PMC4763867

[112] Renzi TA Rubino M Gornati L Garlanda C Locati M Curtale G. MiR-146b mediates endotoxin tolerance in human phagocytes. Med. Inflamm. (2015) 2015:145305. 10.1155/2015/145305 26451077 PMC4584235

[113] Curtale G Renzi TA Drufuca L Rubino M Locati M. Glucocorticoids downregulate TLR4 signaling activity via its direct targeting by miR-511-5p. Eur J Immun. (2017) 47:2080–9. 10.1002/eji.201747044 28776644

[114] Wu C Xue Y Wang P Lin L Liu Q Li N. IFN-gamma primes macrophage activation by increasing phosphatase and tensin homolog via downregulation of miR-3473b. J Immunol. (2014) 193:3036–44. 10.4049/jimmunol.1302379 25092892

[115] Lee C Mitsialis SA Aslam M Vitali SH Vergadi E Konstantinou G. Exosomes mediate the cytoprotective action of mesenchymal stromal cells on hypoxia-induced pulmonary hypertension. Circulation (2012) 126:2601–11. 10.1161/CIRCULATIONAHA.112.114173 23114789 PMC3979353

[116] Feng Y Zou L Yan D Chen H Xu G Jian W. Extracellular microRNAs induce potent innate immune responses via TLR7/MyD88-dependent mechanisms. J Immunol. (2017) 199:2106–17. 10.4049/jimmunol.1700730 28768728 PMC6462263

[117] Willis GR Mitsialis SA Kourembanas S. Good things come in small packages: application of exosome-based therapeutics in neonatal lung injury. Pediatr. Res. (2018) 83:298–307. 10.1038/pr.2017.256 28985201 PMC5876073

[118] Fabbri M. TLRs as miRNA receptors. Cancer Res. (2012) 72:6333–7. 10.1158/0008-5472.CAN-12-3229 23222301

[119] Tung SL Boardman DA Sen M Letizia M Peng Q Cianci N. Regulatory T cell-derived extracellular vesicles modify dendritic cell function. Sci Rep. (2018) 8:6065. 10.1038/s41598-018-24531-8 29666503 PMC5904112

